# The association of fracture risk in atrial fibrillation patients and long-term anticoagulant therapy category: a systematic review and meta-analysis

**DOI:** 10.7717/peerj.10683

**Published:** 2021-01-25

**Authors:** Jun Chen, Lingchun Lyu, Jiayi Shen, Chunlai Zeng, Cheng Chen, Tiemin Wei

**Affiliations:** 1 Medical College of Zhejiang University, Hangzhou, Zhejiang, China; 2 Lishui Hospital of Zhejiang University School of Medicine, Lishui, Zhejiang, China

**Keywords:** Atrial fibrillation, Non-vitamin K antagonist oral anticoagulants, Warfarin, Fracture, Meta-analysis

## Abstract

**Objective:**

Our study aimed to assess the risk of all fractures and hip fractures in patients with atrial fibrillation (AF) who took non-vitamin K antagonist oral anticoagulants (NOACs) compared to warfarin.

**Methods:**

We searched PubMed, Embase, and Cochrane Library and Clinical Trials.gov Website. Reviewed related researches up to January 31, 2020, to identify studies with more than 12 months of follow-up data. The protocol for this systematic review and meta-analysis has been registered in the International Prospective Register of Systematic Reviews (PROSPERO Number: CRD42020156893).

**Results:**

We included five RCT studies, and five observational studies that contained a total of 326,846 patients in our meta-analysis. Our meta-analysis showed that patients taken NOACs had no significant all fracture risk (RR = 0.91, 95% CI [0.81–1.01]) and hip fracture risk (RR = 0.92, 95% CI [0.82–1.03]) compared with those taken warfarin. Subanalysis showed that the risk of all fractures and hip fractures treated by NOACs were significant lower compared with warfarin in observational studies compared with RCT studies. Also, a subanalysis across the duration of anticoagulation showed the NOACs users have lower all fracture risk than warfarin users when the duration of anticoagulation ≤2 years (RR = 0.89, 95% CI [0.80–0.99]). Further analysis, significant lower all fracture risk in the rivaroxaban therapy (RR = 0.81; 95% CI [0.76–0.86]) compared with warfarin but no statistical significance in hip fracture. There were no significant difference of all fracture risk and hip fracture risk in dabigatran, apixaban, and edoxaban therapy compared with warfarin.

**Conclusion:**

The meta-analysis demonstrated that NOACs associated with a significantly lower all fracture risk compared with warfarin when the duration of anticoagulation more than 2 years. Rivaroxaban users had lower risk of all fracture than warfarin users in AF patients. But there was no evidence to verify apixaban, edoxaban, and dabigatranin could decrease all fracture and hip fracture risk compared with warfarin.

## Introduction

Atrial fibrillation (AF) is the most common type of cardiac arrhythmia, and is responsible for increased cardiovascular and cerebrovascular disorders throughout the world. This has led to significantly higher health care costs and a global public health burden (Wendelboe & Raskob, 2016). It is particularly prevalent among the elderly, and epidemiological studies have consistently found that the prevalence of AF gradually increases with age (Staerk et al., 2017). It is estimated that 6–12 million people will develop AF in the US by 2050 and 17.9 million people in Europe by 2060 (Lippi, Sanchis-Gomar & Cervellin, 2020). As the improvement of atrial fibrillation related guidelines, guideline-directed management and therapy play an important role in the prevention of strokes and other viscera embolisms in AF patients. Anticoagulation therapy is the main strategy for long-term treatment of non-valvular AF to prevent strokes and other important organ embolisms (January et al., 2019). Warfarin, a vitamin K antagonist, has been the standard anticoagulant treatment for AF for decades (Morais & De Caterina, 2016). Gage et al. (2006) reported, however, that long-term use of warfarin is associated with a higher risk of osteoporotic fractures (odds ratio, OR: 1.25; 95% CI [1.06–1.48], *P* = 0.03) (Gage et al., 2006). This study has aroused great interest from cardiologists, because it sheds light on non-bleeding adverse events associated with oral anticoagulant treatments for AF patients.

Osteoporosis and fractures, especially hip fractures, are common among the elderly, with an age-adjusted incidence rate of at least 150–250 per 100,000 people worldwide. Hip fractures not only affect quality of life, but they increase the risk of mortality in the next twenty years (Collin et al., 2017). Recently, several retrospective studies have suggested that NOACs reduce the risk of fractures in patients with AF compared to warfarin (Binding et al., 2019; Huang et al., 2020a; Lau et al., 2017; Lutsey et al., 2020). However, hip fracture risks in AF patients between warfarin therapy and NOACs therapy even specific NOACs therapy was not clear. The association between hip fracture risks in AF patients and specific anticoagulant therapy needs to be explored further because of the application potential of oral anticoagulants for AF patients. To clarify this question, we conducted a systematic review and meta-analysis to assess the risk of fractures for AF patients treated with NOACs versus warfarin.

## Materials & Methods

This systematic review was conducted in accordance with the Cochrane Collaboration Handbook, Observational Studies in Epidemiology Statement (Tu & Greenwood, 2012), the Meta-Analysis and Systematic Reviews of Observational Studies (Moher et al., 2009), and the Preferred Reporting Items for Systematic review and Meta-Analysis (PRISMA) (Moher et al., 2009). The Preferred Reporting Items for Systematic review and Meta-Analysis Protocols (PRISMA-P) are shown in File S1. The protocol for this systematic review and meta-analysis was registered in the International Prospective Register of Systematic Reviews (PROSPERO, CRD 42020156893).

### Search strategy

**Figure 1:**
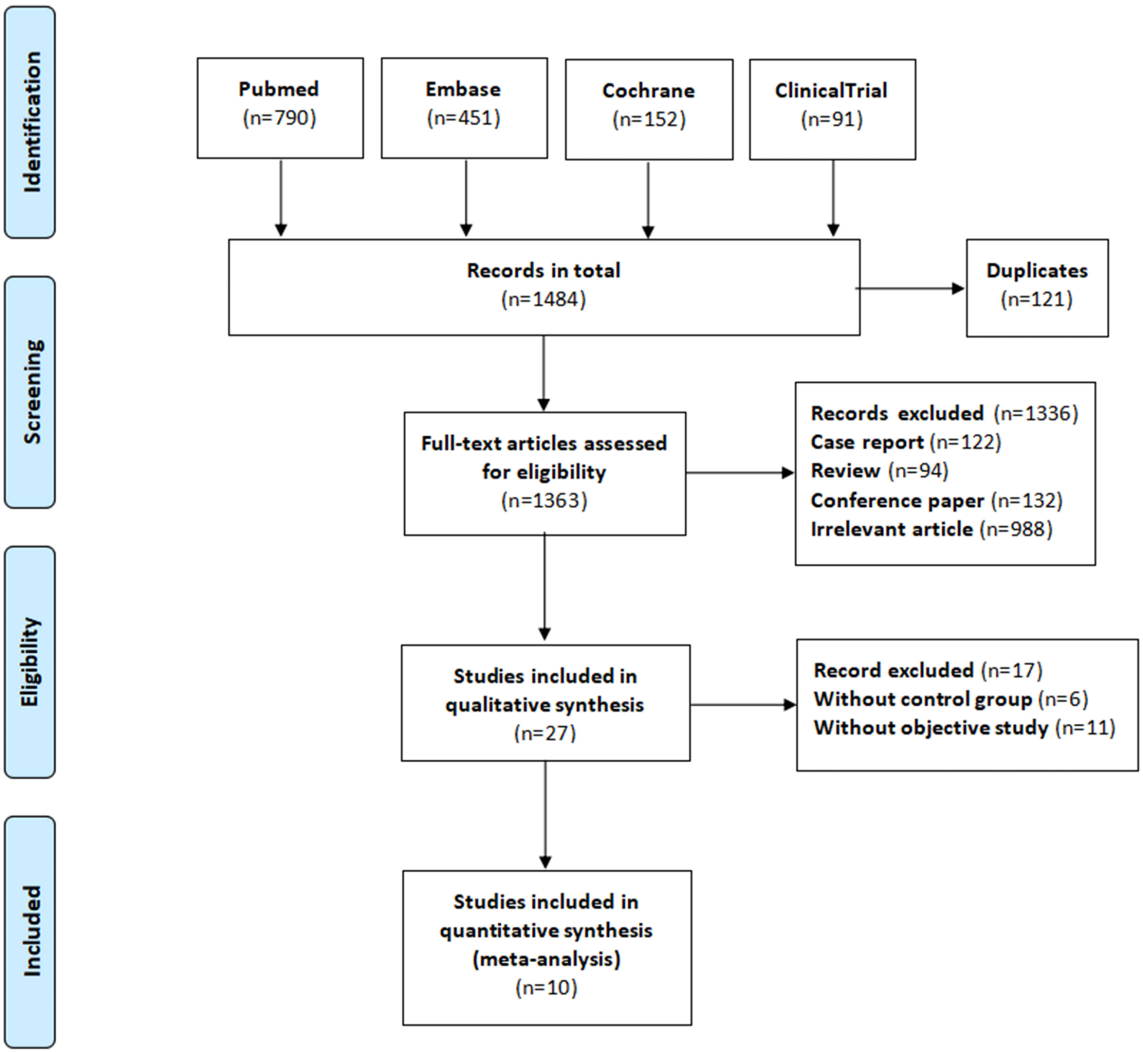
The process of study selection.

We searched the PubMed, Embase, and Cochrane Library databases, as well as the Clinical Trials.gov website, until January 31, 2020. The search keywords included “atrial fibrillation,” “anticoagulant,” “direct oral anticoagulant,” “vitamin K antagonist oral anticoagulants,” “non-vitamin K antagonist oral anticoagulants,” “dabigatran,” “rivaroxaban,” “apixaban,” “edoxaban,” “warfarin,” “fracture,” “osteoporosis,” “osteoporotic fractures,” and “rarefaction of bone.” We did not include any language restrictions. Our detailed search strategy is shown in File S2. The reference list for each included study was reviewed, and potentially related research from these reference lists was manually searched. Our systematic review was completed in accordance with the guidelines for the Preferred Reporting Items for Systematic Reviews and Meta-Analyses (PRISMA) (Moher et al., 2009), as shown in Fig. 1.

### Inclusion and exclusion criteria

The inclusion criteria for the research studies to be analyzed were the following: (1) study patients diagnosed with AF above the age of 18 years and taking oral anticoagulants due to a diagnosis of AF; (2) the study contained at least two comparison groups, one receiving warfarin therapy and the other receiving one of the NOACs (e.g., dabigatran, rivaroxaban, apixaban, edoxaban); (3) considering that the duration of the anticoagulant therapy was not uniform for each patient in the included studies, we determined that the follow-up time for the included studies must exceed 12 months to ensure that each patient received long-term anticoagulant therapy; and (4) the fracture events (e.g., all-fracture and hip fracture) were reported or documented on the Clinical Trials.gov website. The exclusion criteria were as follows: (1) for duplicated reports, the article that was published last was selected; (2) patients administered long-term oral anticoagulant therapy for reasons other than AF; (3) reports that are reviews, case reports, or unrelated studies; (4) the outcome data was unavailable.

### Study selection and data extraction

All the studies were independently assessed by two reviewers (SJY and CC). After excluding duplicates, the remaining studies were read to identify potentially suitable articles by title and abstract. The reviewers independently assessed these studies for eligibility. Disagreements were resolved by discussion with a third reviewer (LLC). Data were extracted from each included article, including authors, study type, publication date, follow-up duration, study size, baseline characteristics of each study population, study interventions, and the principal summary measure (e.g., adjusted risk ratio [RR], adjusted hazard ratio [HR], and adjusted odds ratio). Relevant outcomes included all-fractures and hip fractures.

### Study quality assessment

The Cochrane Collaboration tool was used to assess the risk of bias in the randomized controlled trials (RCTs) (Higgins et al., 2011). The assessment included the generation of random sequences, blinding of patients and researchers, allocation concealment, blinding of the outcome assessment, selective reporting of outcomes, completeness of the outcome data, and other threats to the validity of the results. The Newcastle Ottawa quality assessment scale (NOS) (Higgins et al., 2011) was used to evaluate the quality of the observational studies. The assessment factors included the selection of the study groups, comparability of the groups, and the ascertainment of the exposure and outcomes. A score of 7 or more was considered high-quality.

### Data synthesis and analysis

Our main outcomes were all-fracture and hip fracture risks in NOAC users versus warfarin users. We used RRs and their associated 95% confidence intervals (CI) to assess the outcomes, and a *P* value < 0.05 was considered statistically significant. The meta-analysis heterogeneity was quantified with the *I*^2^ test (low: 0–20%; moderate: 20–50%; and high: >50%). RR and their 95% confidence intervals (CIs) were calculated using random-effect models. We performed two meta-analyses and pooled the risk of all-fractures and hip fractures for NOAC versus warfarin users and stratified the results by study type (observational studies or RCTs), and duration of anticoagulation treatment (≥2 years or <2 years), respectively. We performed a subgroup analysis to evaluate the risk of all-fractures and hip fractures for each specific NOAC compared to warfarin. We also performed a sensitivity analysis to assess the impact of each study on the overall results by deleting individual articles and meta-merging the remaining ones. Publication bias within the results was displayed using a funnel plot. We used Review Manager version 5.3 (The Nordic Cochrane Centre, The Cochrane Collaboration, Copenhagen, Denmark) and Stata 14.0 (StataCorp, College Station, TX, USA) to compile our data and perform the meta-analysis.

## Results

### Inclusion of studies and quality assessment

Our database search yielded 1,484 research articles and clinical trials. After removing duplicates, reviews, case reports, unrelated studies, and those with unavailable data, 10 articles and 12 datasets were remained to include in our meta-analysis. Five of the articles were observational studies, and the other five were RCT studies with double-blind designs (Table 1). The overall process is illustrated in Fig. 1. The five observational studies were all retrospective cohort studies that directly compared the fracture risk for NOAC users with warfarin users. None of the RCT studies reported fracture events in the articles themselves, but they did document the outcomes on the Clinical Trials.gov Website. We counted the fracture events from each of these studies and calculated the RR values. The outcome data and RR values of each RCT study are documented in Tables S1 and S2 (File S4). The baseline characteristics of the patients are documented in Table 2. The mean age of the patients in each study ranged from 67 to 74 years. Regarding the total number of patients reported on in these studies, 12.4–63.4% of the study population had a history of stroke or transient ischemic attack. Diabetes and heart failure were common in the included studies, accounting for 13.8–40.4% and 17.3–62.3% of the study patients, respectively. Additionally, 30.5–40.5% of the study patients received aspirin antiplatelet therapy. All five of the RCT studies were considered high-quality because of their low risk of bias. Of the five observational studies, four were considered high-quality for their representativeness of the exposed cohort, sufficient follow-up for reliable outcomes, and low risk of bias, while the remaining one was considered a medium-quality study due to an insufficient baseline in the study population, significant clinical heterogeneity, and a lack of adjustment for confounding variables. The results of the quality assessment for the studies is shown in Table 1. For additional details, please refer to Tables S3 and S4 (File S4).

**Table 1:** Characteristics of studies include.

***Author /year***	***Size***	***Follow-up***	***Age***	***Study type***	***Interventions (no.)***	***Comparisons***	***Outcomes***	***Measures***	***Risk of bias***
**Connolly/2009**	**18,113**	**2.0 y**	**71 y**	**RCT**	**Dabigatran(110 mg) (*n* = 6015)**	**NOACs vs warfarin**	**all fracture,**	**risk ratio**	**low risk**
**(NCT00262600)**					**Dabigatran(150 mg) (*n* = 6076)**	**dabigatran(110 mg) vs warfarin**	**hip fracture,**		
					**warfarin (*n* = 6022)**	**dabigatran(150 mg) vs warfarin**	**vertebral fracture**		
**Patel/2011**	**14,264**	**1.9 y**	**73 y**	**RCT**	**Rivaroxaban(20 mg) (*n* = 7131)**	**NOACs vs warfarin**	**all fracture,**	**risk ratio**	**low risk**
**(NCT00403767)**					**warfarin (*n* = 7133)**	**rivaroxaban(20 mg) vs warfarin**	**hip fracture,**		
							**vertebral fracture**		
**Hori et al.,2012**	**1,278**	**1.5y**	**71 y**	**RCT**	**rivaroxaban (15 mg) (*n* = 639)**	**NOACs vs warfarin**	**all fracture,**	**risk ratio**	**low risk**
**(NCT00494871)**					**warfarin (*n* = 639)**	**rivaroxaban(15 mg) vs warfarin**	**hip fracture,**		
							**vertebral fracture**		
**Granger/2013**	**18,201**	**1.8 y**	**70 y**	**RCT**	**apixaban (5 mg) (*n* = 9120)**	**NOACs vs warfarin**	**all fracture,**	**risk ratio**	**low risk**
**(NCT00412984)**					**warfarin (*n* = 9081)**	**Apixaban(5 mg) vs warfarin**	**hip fracture,**		
							**vertebral fracture**		
**Giugliano/2013**	**21,105**	**2.8 y**	**72 y**	**RCT**	**edoxaban (60 mg) (*n* = 7035)**	**NOACs vs warfarin**	**all fracture,**	**risk ratio**	**low risk**
**(NCT00781391)**					**edoxaban (30 mg) (*n* = 7034)**	**edoxaban (60 mg) vs warfarin**	**hip fracture,**		
					**warfarin (*n* = 7036)**	**edoxaban (30 mg) vs warfarin**	**vertebral fracture**		
**Lau/2017**	**10,279**	**1.4 y**	**74 y**	**Retr-cohort**	**dabigatran (*n* = 3, 298)**	**NOACs vs warfarin**	**hip fracture,**	**a IRR**	**low risk**
					**warfarin (*n* = 6, 981)**	**dabigatran vs warfarin**	**vertebral fracture**		
**Binding/2019**	**37,350**	**2.0 y**	**73 y**	**Retr-cohort**	**NOACs (*n* = 25, 182)**	**NOACs vs warfarin**	**all fracture,**	**HR**	**low risk**
					**warfarin (*n* = 12, 168)**		**hip fracture,**		
							**osteoporotic fracture**		
**Lutsey/2019**	**167,275**	**1.4 y**	**68.9 y**	**Retr-cohort**	**dabigatran (*n* = 31, 647)**	**NOACs vs warfarin**	**all fracture,**	**aHR**	**low risk**
					**rivaroxaban (*n* = 35, 252)**	**dabigatran vs warfarin**	**hip fracture,**		
					**apixaban (*n* = 17, 751)**	**rivaroxaban vs warfarin**	**fracture require**		
					**warfarin (*n* = 82, 625)**	**apixaban vs warfarin**	**hospitalization**		
**Huang/2020**	**22,131**	**2.4 y**	**72 y**	**Retr-cohort**	**dabigatran (*n* = 5, 796)**	**NOACs vs warfarin**	**all fracture,**	**aHR**	**low risk**
					**rivaroxaban (*n* = 7, 287)**	**dabigatran vs warfarin**	**hip fracture,**		
					**apixaban (*n* = 1, 761)**	**rivaroxaban vs warfarin**	**vertebral fracture**		
					**warfarin (*n* = 7, 287)**	**apixaban vs warfarin**	**Humerus/forearm/ wrist fractures**		
**Lucenteforte et al., 2017**	**16,850**	**1.0 y**	**71 y**	**Retr-cohort**	**NOACs (*n* = 2474)**	**NOACs vs warfarin**	**all fracture**	**aHR**	**medium**
					**dabigatran (*n* = 1, 285)**	**dabigatran vs warfarin**			**risk**
					**warfarin (*n* = 13, 091)**				

**Notes.**

aHR Adjusted hazard ratio a IRR Adjusted incidence rate ratio Retr-cohort Retrospective cohort

**Table 2:** Characteristics of included studies patients

**Author /year**	**Connolly/2009**			**Patel/2011**		**Granger/2013**		**Hori/2012**		**Lau/2017**	
**Characteristic**	**Dabigatran 110 mg**	**Dabigatran 150 mg**	**Warfarin**	**Rivaroxaban**	**Warfarin**	**Apixaban**	**Warfarin**	**Rivaroxaban**	**Warfarin**	**Dabigatran**	**Warfarin**
**Number**	**6,015**	**6,076**	**6,022**	**7,131**	**7,133**	**9,120**	**9,081**	**639**	**639**	**3,268**	**4,884**
**Age-years ± SD**	**71.4 ± 8.6**	**71.5 ± 8.8**	**71.6 ± 8.6**	**73**	**73**	**70**	**70**	**71.0**	**71.2**	**74.2 ± 10.1**	**73.3 ± 11.0**
**Male (%)**	**3,868(64.3)**	**3,840(63.2)**	**3,812(63.3)**	**4,300(60.3)**	**4,301(60.3)**	**5,882(64.5)**	**5,903(65)**	**530(82.9)**	**500(78.2)**	**1,611(49.3)**	**2,489(51)**
**CHA2DS2/CHA2DS2-VASc score**	**2.1 ± 1.1**	**2.2 ± 1.2**	**2.1 ± 1.1**	**3.48 ± 0.94**	**3.46 ± 0.95**	**2.1 ± 1.1**	**2.1 ± 1.1**	**3.27**	**3.22**	**2.1 ± 1.5**	**2.1 ± 1.6**
**TIA/Stroke history (%)**	**1,197(19.9)**	**1,233(20.3)**	**1,192(19.8)**	**3,915(54.9)**	**3,895(54.6)**	**1,751(19.2)**	**1,789(19.7)**	**408(63.8)**	**405(63.4)**	**1,094(33.5)**	**1,515(31.0)**
**Heart failure (%)**	**1,937(32.2)**	**1,932(31.8)**	**1,921(31.9)**	**4,464(62.6)**	**4,444(62.3)**	**3,238(35.5)**	**3,215(35.4)**	**264(41.3)**	**257(40.2)**	**690(21.1)**	**1,270(26.0)**
**Myocardial infarction (%)**	**1,011(16.8)**	**1,027(16.9)**	**970(16.1)**	**1,184(16.6)**	**1,284(18.0)**	**1,322(14.5)**	**1,262(13.9)**	**45(7.0)**	**53(8.3)**	**NA**	**NA**
**Diabetes (%)**	**1,408(23.4)**	**1,404(23.1)**	**1,409(23.4)**	**2,881(40.4)**	**2,818(39.5)**	**2,280(25.0)**	**2,261(24.9)**	**249(39.0)**	**237(37.1)**	**984(30.1)**	**1,402(28.7)**
**prior fractures (%)**	**NA**	**NA**	**NA**	**NA**	**NA**	**NA**	**NA**	**NA**	**NA**	**234(7.2)**	**336(6.9)**
**Osteoporosis (%)**	**NA**	**NA**	**NA**	**NA**	**NA**	**NA**	**NA**	**NA**	**NA**	**38(1.2)**	**53(1.1)**
**COPD (%)**	**NA**	**NA**	**NA**	**756(10.6)**	**742(10.4)**	**NA**	**NA**	**NA**	**NA**	**270(8.3)**	**406(8.3)**
**CKD (%)**	**NA**	**NA**	**NA**	**NA**	**NA**	**NA**	**NA**	**NA**	**NA**	**94(2.9)**	**181(3.7)**
**Aspirin (%)**	**2,406(40.0)**	**2,351(38.7)**	**40.6**	**2,589(36.3)**	**2,618(36.7)**	**2,855(31.3)**	**2,770(30.5)**	**NA**	**NA**	**NA**	**NA**

Author/year	Binding/2019		Lutsey/2019				Huang/2020		Giugliano/2013			Lucenteforte/2017		
**Characteristic**	**NOACs**	**Warfarin**	**Dabigatran**	**Rivaroxaban**	**Apixaban**	**Warfarin**	**NOACs**	**Warfarin**	**Edoxaban60 mg**	**Edoxaban30 mg**	**Warfarin**	**NOACs**	**dabigatran**	**Warfarin**
**Number**	**25,182**	**12,168**	**31,647**	**35,252**	**17,751**	**82,625**	**9707**	**9707**	**7,035**	**7,034**	**7,036**	**2,474**	**1,285**	**13,091**
**Age- years ±SD**	**73 ± 7.0**	**72+7.0**	**67.0 ± 12.4**	**67.7 ± 12.3**	**69.1 ± 12.6**	**70.2 ± 12.3**	**72.4 ± 10.7**	**71.3 ± 11.5**	**72 ± 7**	**72 ± 7**	**72 ± 7**	**NA**	**NA**	**NA**
**Male (%)**	**14,081(55.9)**	**7,515(61.8)**	**20,602(65.1)**	**21,821(61.9)**	**10,668(60.1)**	**50,566(61.2)**	**5,749(59.2)**	**5,714(58.9)**	**4,369(62.1)**	**4,305(61.2)**	**4,398(62.5)**	**1,195**(48.3)	**644**(50.1)	**6,768**(51.7)
**CHA2DS2/CHA2DS2-VASc score**	**NA**	**NA**	**3.0 ± 1.9**	**3.1 ± 1.9**	**3.4 ± 2.0**	**3.6 ± 2.0**	**2.8 ± 1.6**	**2.7 ± 1.8**	**2.8 ± 1.0**	**2.8 ± 1.0**	**2.8 ± 1.0**	**NA**	**NA**	**NA**
**TIA/Stroke history (%)**	**4,420(17.6)**	**1,503(12.4)**	**5,412(17.1)**	**6,063(17.2)**	**3,532(19.9)**	**18,839(22.8)**	**2,662(27.4)**	**2,509(25.9)**	**1,977(28.1)**	**2,005(28.5)**	**1,991(28.3)**	**NA**	**NA**	**NA**
**Heart failure (%)**	**4,367(17.3)**	**2,235(18.4)**	**7,532(23.8)**	**8,672(24.6)**	**4,953(27.9)**	**27,266(33.0)**	**2,551(26.3)**	**2,758(28.4)**	**4,094(58.2)**	**3,981(56.6)**	**4,046(57.5)**	**NA**	**NA**	**NA**
**Myocardial infarction (%)**	**NA**	**NA**	**2,279(7.2)**	**2,891(8.2)**	**1,633(9.2)**	**9,254(11.2)**	**NA**	**NA**	**NA**	**NA**	**NA**	**NA**	**NA**	**NA**
**Diabetes (%)**	**3,480(13.8)**	**1,713(14.1)**	**8,766(27.7)**	**9,941(28.2)**	**5,379(30.3)**	**27,432(33.2)**	**2,485(25.6)**	**2,513(25.9)**	**2,561(36.4)**	**2,546(36.2)**	**2,519(35.8)**	**NA**	**NA**	**NA**
**prior fractures (%)**	**1,038(4.1)**	**347(2.9)**	**1,076(3.4)**	**1,410(4.0)**	**746(4.2)**	**3,553(4.3)**	**NA**	**NA**	**NA**	**NA**	**NA**	**NA**	**NA**	**NA**
**Osteoporosis (%)**	**NA**	**NA**	**8,260(26.1)**	**10,505(29.8)**	**5,006(28.2)**	**24,705(29.9)**	**50(0.5)**	**65(0.7)**	**NA**	**NA**	**NA**	**NA**	**NA**	**NA**
**COPD (%)**	**2,468 (9.8)**	**1,114(9.2)**	**7,026(22.2)**	**8,778(24.9)**	**4,704(26.5)**	**23,383(28.3)**	**801(8.3)**	**787(8.1)**	**NA**	**NA**	**NA**	**NA**	**NA**	**NA**
**CKD (%)**	**NA**	**NA**	**2,215(7.0)**	**2,891(8.2)**	**2,112(11.9)**	**12,063(14.6)**	**652(6.7)**	**762(7.9)**	**NA**	**NA**	**NA**	**NA**	**NA**	**NA**
**Aspirin (%)**	**2,365(9.4)**	**1,947(16.0)**	**NA**	**NA**	**NA**	**NA**	**NA**	**NA**	**2,068(29.4)**	**2,019(28.7)**	**2,090(29.7)**	**NA**	**NA**	**NA**

### Results of the meta-analysis

**Figure 2:**
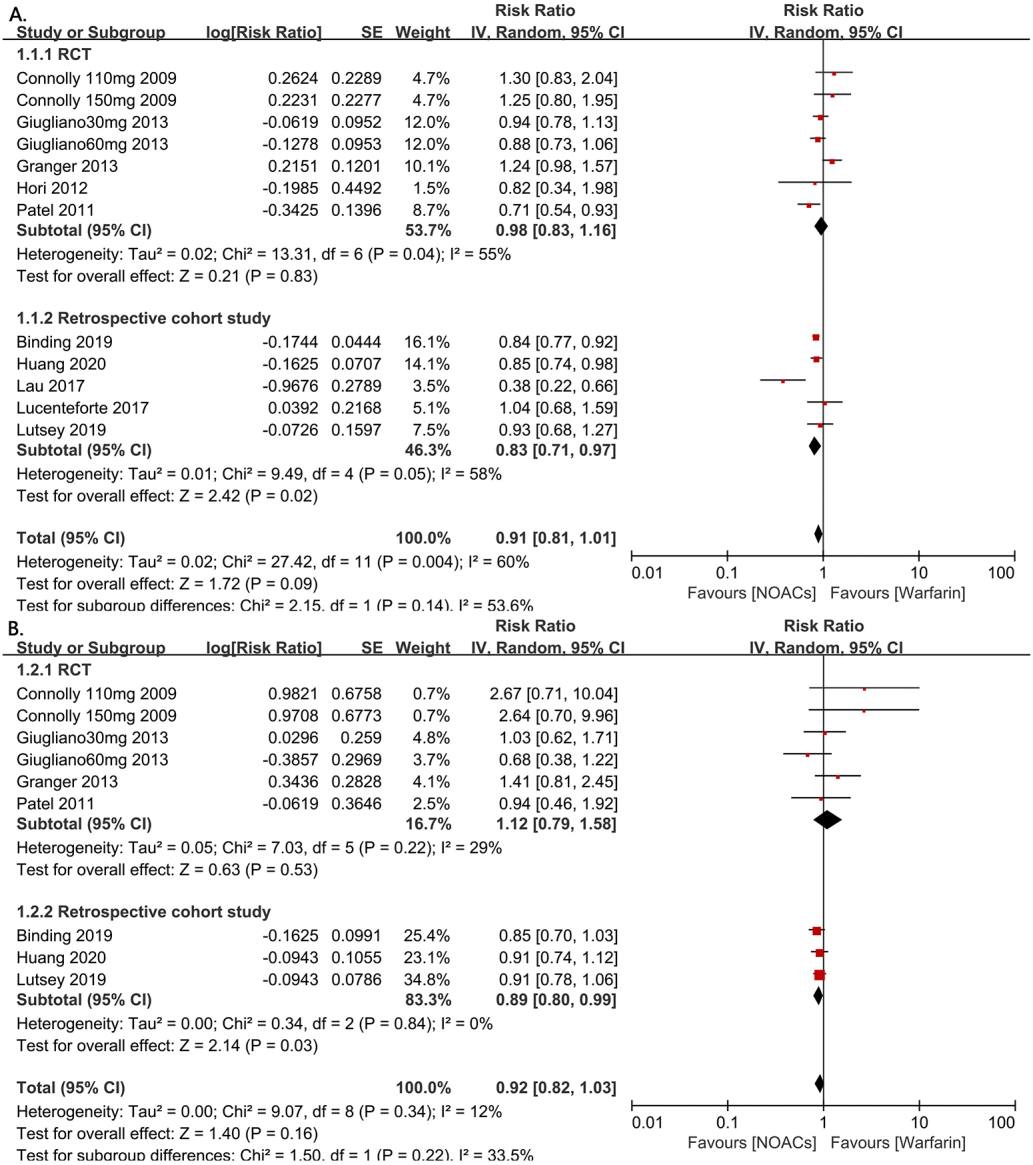
The forest plot of the all-fracture and hip fracture risks for NOACs versus warfarin stratified by study type (observational studies or RCTs). (A) All-fracture risk for NOACs versus warfarin. (B) Hip fracture risk for NOACs versus warfarin. Relative risk (RR) is used to evaluate the fracture risk. The direction of the forest plot coordinates represents the supported objects, which have a lower fracture risk. The diamond figures indicate the point estimate and the left and right ends of the lines [95% confidence interval, CI]. NOACs: non-vitamin K antagonist oral anticoagulants. All of the merge is conducted by random effect model.

The 326,846 subjects from the included studies were pooled together for the all-fracture risk meta-analysis. Patients who were treated with NOACs did not have a significant risk of fracture compared with those taking warfarin (RR=0.91, 95% CI [0.81–1.01], *I*^2^ = 60%, Fig. 2A). Due to the underlying heterogeneity of the article types, however, we analyzed the data in subgroups. Compared with RCTs, those with NOACs therapy had significant lower all fracture risk in retrospective cohort studies (RR = 0.83, 95% CI [0.71–0.97], *P* = 0.02, *I*^2^ = 58%, forest plot shown in Fig. 2 [A]). For the hip fracture risk meta-analysis, data from four RCT studies and three retrospective cohort studies (298,439 patients) were pooled together. In this grouping, there were no differences in the hip fracture risk observed between the NOAC and warfarin groups in these studies, but the patients in the retrospective cohort studies showed a statistically significant decrease in the risk of hip fracture compared to the patients in the RCT studies (RR = 0.89, 95% CI [0.80–0.99], *P* = 0.03, *I*^2^ = 0%, forest plot shown in Fig. 2B).

**Figure 3:**
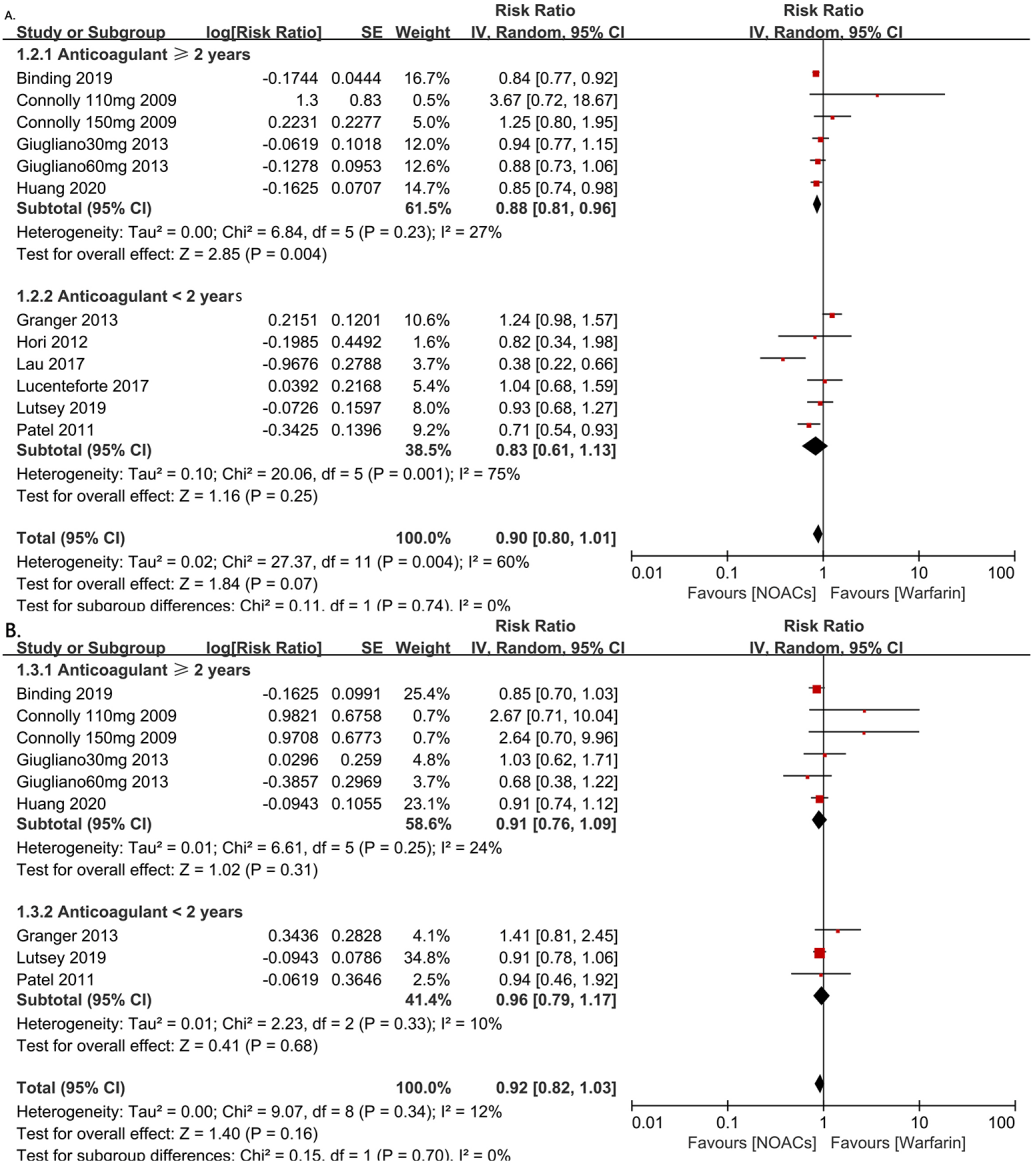
The forest plot of the all-fracture and hip fracture risks for NOACs versus warfarin stratified by duration of anticoagulant treatment (≥2 years or <2 years). (A) All-fracture risk for NOACs versus warfarin. (B) hip fracture risk for NOACs versus warfarin. Relative risk (RR) is used to evaluate the fracture risk. The direction of the forest plot coordinates represents the supported objects, which have a lower fracture risk. The diamond figures indicate the point estimate and the left and right ends of the lines [95% confidence interval, CI]. NOACs: non-vitamin K antagonist oral anticoagulants. All of the merges are conducted by a random effect model.

To investigate the influence of anticoagulant treatment duration on the risk of all-fractures and hip fractures, we regrouped the studies based on whether the anticoagulant therapies were administered for <2 years or ≥2 years. The results showed the NOACs users have a lower all-fracture risk than warfarin users when the anticoagulation therapy duration was ≥2 years (4 RCTs and 2 observational studies, RR = 0.88, 95% CI [0.81–0.96], *P* = 0.004, *I*^2^ = 27%, forest plot shown in Fig. 3A), while no differences in hip fracture risk.

**Figure 4:**
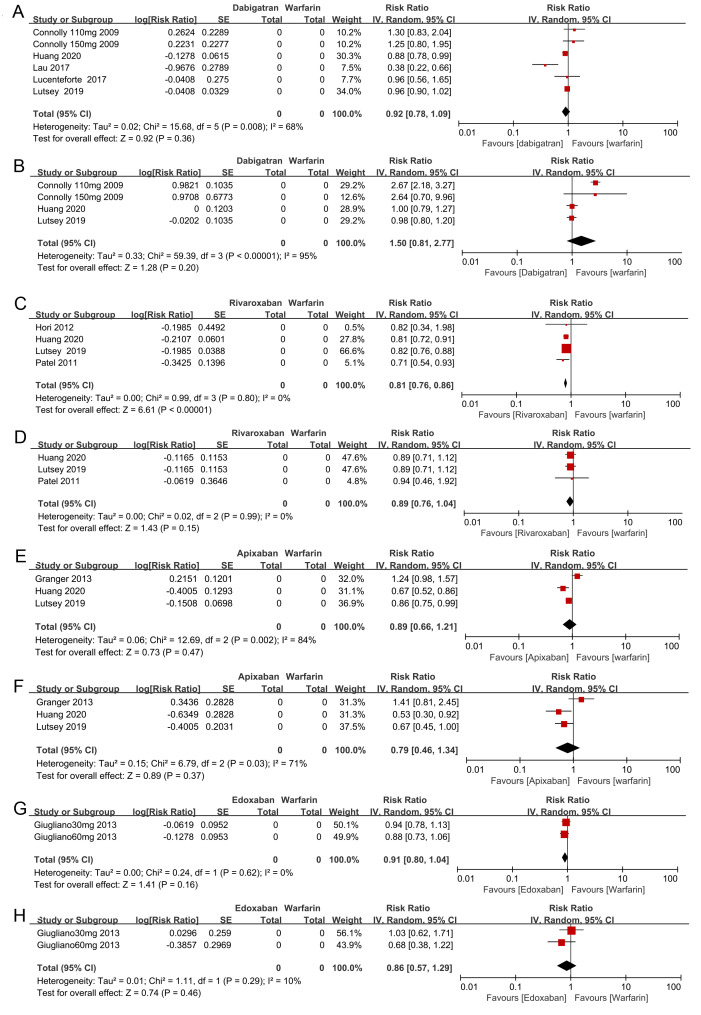
The forest plot of the all-fracture and hip fracture risks for each subgroup of NOACs versus warfarin. (A) All-fracture risk of dabigatran versus warfarin; (B) hip fracture risk of dabigatran versus warfarin; (C) all-fracture risk of rivaroxaban versus warfarin; (D) hip fracture risk of rivaroxaban versus warfarin; (E) all-fracture risk of apixaban versus warfarin; (F) hip fracture risk of apixaban versus warfarin; (G) all-fracture risk of edoxaban versus warfarin; (H) hip fracture risk of edoxaban versus warfarin.

We also performed another subgroup analysis in which we compared the all-fracture and hip fracture risks for each of the individual NOACs (e.g., dabigatran, rivaroxaban, apixaban, and edoxaban) to warfarin. We included 2 of the observational studies and 2 RCTs in this analysis. There was insufficient evidence to prove that dabigatran, apixaban, and edoxaban were associated with lower all-fracture or hip fracture risks compared with warfarin (see the forest plot shown in Figs. 4A, 4B, 4E, 4F, 4G, 4H). However, rivaroxaban anticoagulant therapy patients did show a significantly lower risk of all-fractures than warfarin patients (two observational studies and two RCTs, RR = 0.81, 95% CI [0.76–0.86], *I*^2^ = 0%, Fig. 4C), but rivaroxaban anticoagulant therapy failed to significantly decrease the hip fracture risk (RR = 0.89, 95% CI [0.76–1.04], *I*^2^ = 0%, Fig. 4D).

**Figure 5:**
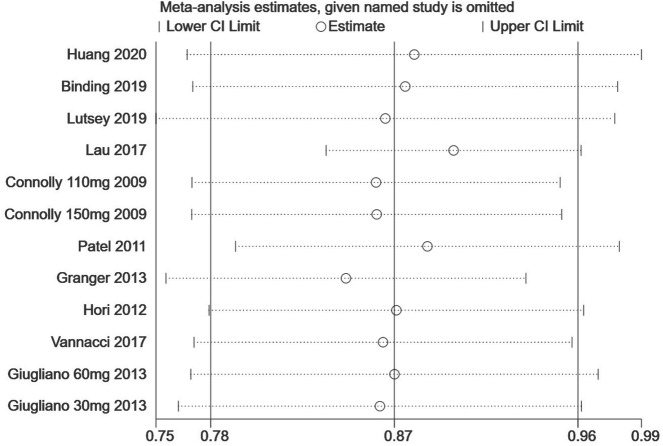
The sensitivity analysis for the all-fracture risk of NOACs versus warfarin. Each branch represents the named study that was omitted; the merged effect size of the studies that remained.

**Figure 6:**
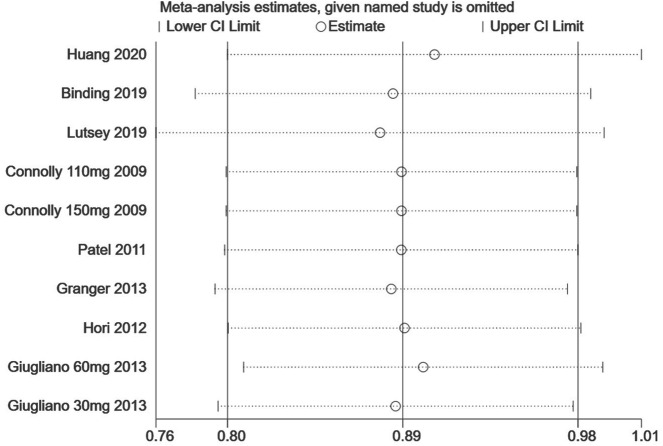
The sensitivity analysis for the hip fracture risk of NOACs versus warfarin.

**Figure 7:**
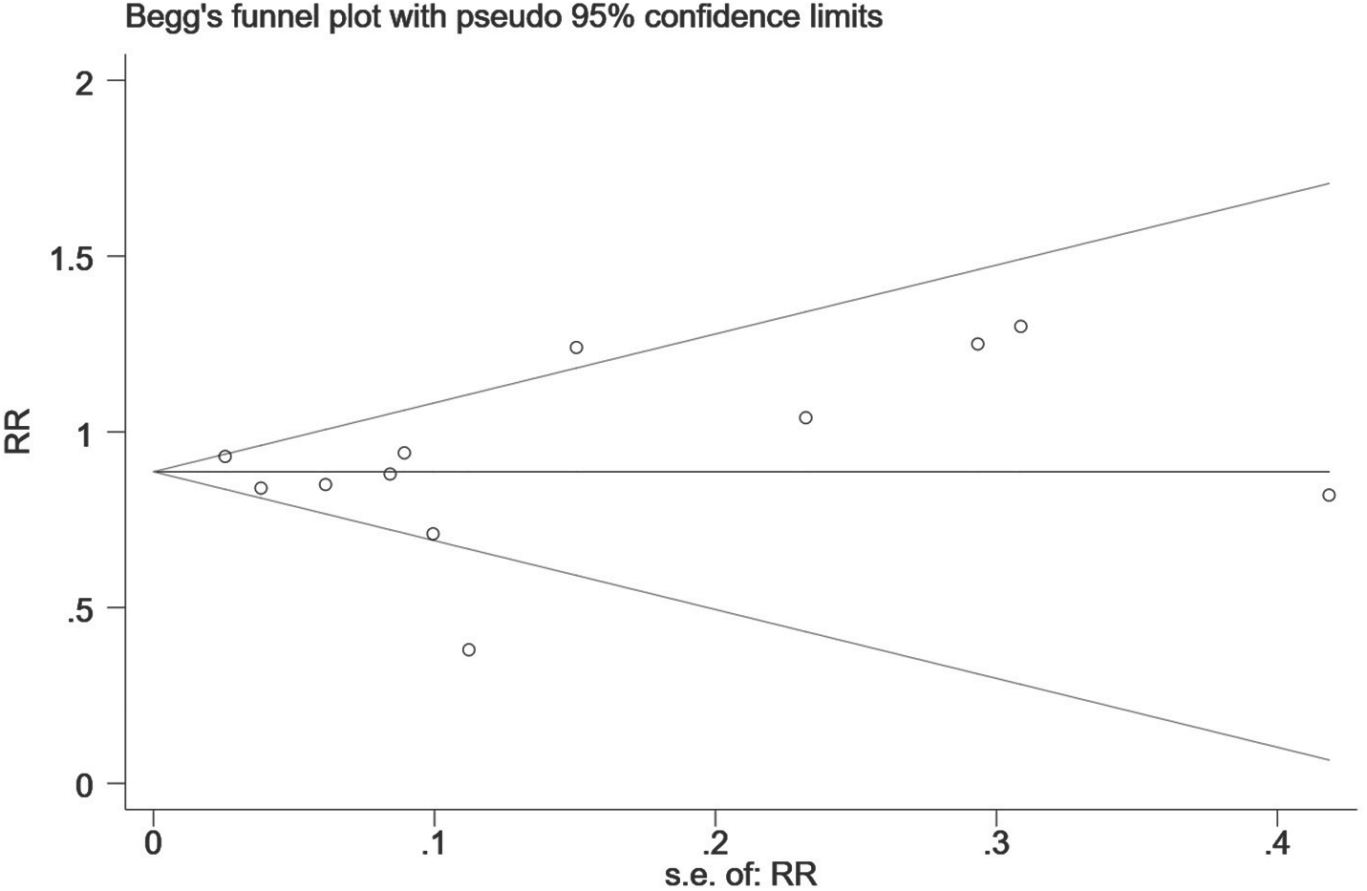
The publication bias for the all-fracture risk of NOACs versus warfarin. The horizontal line represents the merged effect size of the fracture risk and the funnel sample lines represent the 95% confidence interval value. Each hollow point represents each included study. The publication bias is determined by checking the distribution of each hollow point, regardless of whether it is symmetrical. The symmetrical distribution represents publication bias that is not obvious.

**Figure 8:**
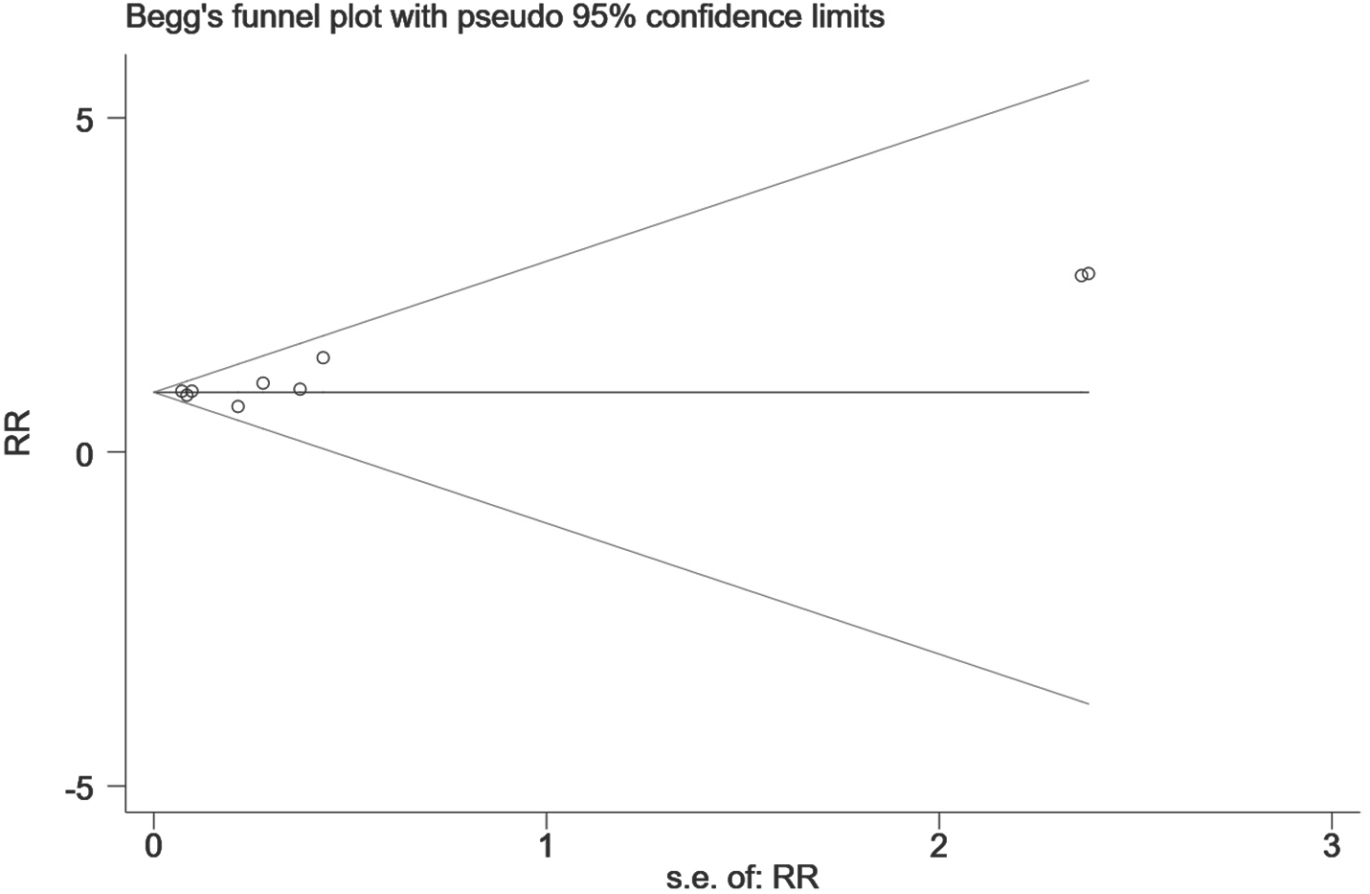
The publication bias for the hip fracture risk of NOACs versus warfarin.

Finally, we performed a sensitivity analysis to test the robustness of our results by orderly elimination of each included study and meta merged the rest studies. Our results are statistically reliable, as shown in Figs. 5 and 6. The robust results from this analysis of each NOAC is shown in Fig. S1. The publication bias was then assessed using a funnel plot. As shown in Figs. 7 and 8, there was no evidence of obvious publication bias in our study, but a slight bias brought by some grey literature cannot be completely excluded in our study.

## Discussion

Our meta-analysis was based on 10 pooled studies (five large randomized clinical trials and five observational studies) that included 326,846 patients with AF who underwent long-term anticoagulant treatment. To our knowledge, this is the largest systematic review to evaluate the fracture risks of patients with AF taking chronic NOACs versus warfarin. Our results show that AF patients who underwent NOAC treatment for more than 2 years had a 12% lower relative fracture risk than AF patients treated with warfarin. Further analyses demonstrate that, although apixaban, edoxaban, and dabigatran have no effect on all-fracture or hip fracture risks, rivaroxaban does show a strong protective effect against all-fractures in patients with AF, significantly reducing the risk by 19% compared with warfarin. It shows no effect on hip fracture risk, however.

Previous meta-analyses have reported associations between fracture risk and anticoagulant therapies. One study showed a higher risk of incidental hip fractures in vitamin K antagonist (VKA) users than in healthy controls (four studies; RR = 1.17; 95% CI [1.05–1.31]), but there was no increased risk of all-fractures when only the studies matching VKA users with healthy controls (2 studies, RR = 1.03, 95% CI [0.90–1.18]) (Veronese et al., 2015) were analyzed. Veronese et al. also argued that VKAs did not increase prospectively assessed fracture risks compared with healthy controls. However, only two matched studies were pooled for these final results, and they did not report any analyses across study types or durations of anticoagulation therapies in their study. Another meta-analysis conducted by Fiordellisi et al. included 23 articles (22 observational studies and 1 RCT), and they showed that VKA use did not increase the odds of all-fractures or any specific types of fractures (hip, vertebral, wrist, or rib) (Fiordellisi, White & Schweizer, 2019). In their study, Fiordellisi et al. analyzed the subgroup data across study types, fracture types, durations of anticoagulation therapies, age, and gender. Their analyses showed that only the VKA users ≥ 65 years old and female VKA user subgroups demonstrated a significantly increased fracture risk compared with non-VKA users. However, most of the included reports in their study compared VKA users with non-anticoagulant therapy populations; only four reports were of studies directly comparing the risk of fractures in VKA users versus NOAC users. In addition, the reasons for anticoagulant therapy were varied (hemodialysis, systemic lupus erythematosus, etc.).

Our meta-analysis only included studies that patients received oral anticoagulant therapy due to AF. We found that observational studies tended to show that NOAC therapy could significantly decrease all-fracture and hip fracture risks compared to warfarin. Although propensity score matching could have balanced the baseline differences between the warfarin and NOACs users, the choice of anticoagulant therapy for the AF patients was not random. Therapy choices may have been affected by family economic status or the comprehensive condition of the patients. A cross-sectional study reported a higher prevalence of osteoporosis-related fractures among women with a lower economic status (Moradzadeh et al., 2016). Considering there is a trend of warfarin use over NOACs for lower economic status patients, economic levels should be taken into account in future studies.

It is suspected that warfarin impairs normal bone metabolism, resulting in abnormal non-carboxylated or undercarboxylated osteocalcin (Rezaieyazdi et al., 2009). Simon et al. (2002) proposed that warfarin increases osteoclast numbers while decreasing the number and activity of osteoblasts, resulting in bone loss and a reduction in the biomechanical strength of rat femurs. The impact of warfarin on fractures is realized through its cumulative effect, continued warfarin use tends to lead to osteoporosis, which can then lead to fractures. Recently, a large retrospective cohort study found that NOACs are associated with a lower osteoporosis risk compared to warfarin, with rivaroxaban (aHR = 0.68; 95% CI [0.55–0.83]) and apixaban (aHR = 0.38; 95% CI [0.22–0.66]) showing the most significant decreases (Huang et al., 2020b). More importantly, this study found that the association between NOAC use and a lower incidence of osteoporosis seemed to be stronger in those with a longer therapy duration (Huang et al., 2020b). This conclusion was similar to our results showing that the all-fracture risk was lower in NOAC users when the duration of anticoagulation was more than 2 years (2 observational studies and 2 RCTs, RR = 0.88, 95% CI [0.81–0.96], *I*^2^ = 27%, *P* < 0.01), and rivaroxaban specifically was associated with a lower fracture risk than warfarin (2 observational studies and 2 RCTs, RR = 0.81, 95% CI [0.76–0.86], *I*^2^ = 0%, *P* < 0.01). NOACs have been shown to be as effective as warfarin for the prevention of strokes and cardioembolic complications in patients with nonvalvular AF, and they have also shown an association with a lower risk of bleeding events (Connolly et al., 2009; Granger et al., 2011; Patel et al., 2011; Giugliano et al., 2013). In the European Society of Cardiology Guidelines (Steffel et al., 2018), rivaroxaban is recommended for (1) stroke prevention in AF patients, (2) treatment or long-term prevention of recurrent deep vein thrombosis/pulmonary embolisms, (3) venous thromboembolic event prevention after major orthopedic surgery, (4) stroke prevention after percutaneous coronary interventions (with concomitant atrial fibrillation), (5) secondary prevention of atherothrombotic events post-acute coronary syndrome, and (6) secondary prevention of atherothrombotic events in patients with stable coronary artery disease (without AF). For patients with a history of fractures and osteoporosis, or for whom the risk of falling is high, rivaroxaban might be the best option. It is important to note, however, that although rivaroxaban has been approved in Europe (with a reduced dose regimen) for use in patients with severe chronic kidney disease (stage 4, i.e., a creatinine clearance of 15–29 mL/min), it is contraindicated for patients with a mechanical prosthetic valve or moderate to severe mitral stenosis (usually of rheumatic origin) (Steffel et al., 2018).

Not only did we compare the fracture risk between the NOACs in general and warfarin across study types and durations of anticoagulation, we also extended the comparisons to individual NOACs (dabigatran, rivaroxaban, apixaban, and edoxaban) and warfarin. Our findings provide stronger and more comprehensive evidence for the association between fracture risk and the different NOACs. Our sub-analyses of the all-fracture risks associated with an anticoagulation treatment duration of less than 2 years shows high heterogeneity. Among each of the individual NOAC therapies compared with warfarin, however, only dabigatran and apixaban show high heterogeneity. Most of the included studies showed that NOAC therapy was associated with either lower or similar fracture risks compared with warfarin, while only Connolly et al. and Granger et al. showed an obviously higher fracture risk. These inconsistent results were the main reason for the high heterogeneity. As the number of relevant studies increases in the future, the problem of high heterogeneity may be resolved.

### Study limitations

Our study is the largest review and meta-analysis to investigate the risk of fractures in AF patients by comparing warfarin administration with other treatments. However, some limitations should be considered. First, five of the studies evaluated were observational studies. Although a propensity score-matched analysis is a good method for reducing selection bias, it is inevitable that significant clinical heterogeneity still exists. Although common fracture risk factors (history of fractures and osteoporosis) were propensity matched between the NOAC and warfarin groups in some of the analyzed studies, other important factors, such as bone mineral density, serum calcium, and vitamin D levels were not investigated in any of the reports. Future studies should include these indicators to enhance the strength of the studies. Second, lifelong anticoagulation is the standard treatment for AF management. However, the longest follow-up in the included studies was only 2.8 years. The effects of vitamin K antagonists on bone metabolism are cumulative, and the progression from osteoporosis to fractures is a long process. Therefore, extending the follow- up period in future research is necessary. Third, because the research comparing specific NOACs (dabigatran, rivaroxaban, apixaban, and edoxaban) with warfarin is inadequate, we did not perform a subgroup analysis across the study types (observational study or RCT). This also led to high heterogeneity in several of our comparisons.

## Conclusion

Our systematic review and meta-analysis demonstrate that patients with AF treated with NOACs have a significantly lower all-fracture risk, compared with those treated with warfarin, when the duration of the anticoagulant treatment is more than 2 years. In addition, we observed a significantly lower risk of all-fractures in patients treated with rivaroxaban, but not in those treated with dabigatran, apixaban, or edoxaban. There is still a lack of evidence, however, to verify the significant differences we observed in hip fracture risk reductions among patients treated with rivaroxaban, apixaban, dabigatran, and edoxaban. We hope further research will be conducted to provide more information regarding the effects of NOACs on AF.

##  Supplemental Information

10.7717/peerj.10683/supp-1 Supplemental Information 1 PRISMA checklist PRISMA-P (Preferred Reporting Items for Systematic review and Meta-Analysis Protocols) 2015 checklist: recommended items addressed in our systematic review and meta-analysis.

10.7717/peerj.10683/supp-2 Supplemental Information 2 Search strategy

10.7717/peerj.10683/supp-3 Supplemental Information 3 Meta-Analysis Rationale

10.7717/peerj.10683/supp-4 Supplemental Information 4 Quality evaluation scale for prevalence studies

10.7717/peerj.10683/supp-5 Supplemental Information 5 The fractures numbers of each RCTs; the fractures risk of each RCTs (fracture risk ratio of each NOACs vs warfarin); the risk bias of each RCTs; and the risk bias of each Non-RCTs

10.7717/peerj.10683/supp-6 Supplemental Information 6 The sensitivity analysis for all fracture risk and hip fracture risk of each subgroup NOACs versus warfarin (A) The sensitivity analysis for all fracture risk of dabigatran versus warfarin; (B) The sensitivity analysis for hip fracture risk of dabigatran versus warfarin; (C) The sensitivity analysis for all fracture risk of rivaroxaban versus warfarin; (D) The sensitivity analysis for hip fracture risk of rivaroxaban versus warfarin; (E) The sensitivity analysis for all fracture risk of apixaban versus warfarin; (F) The sensitivity analysis for hip fracture risk of apixaban versus warfarin.

## Abbreviations

AF: atrial fibrillation
TIA: transient ischemic attack
NOACs: non-vitamin K antagonist oral anticoagulants
CI: confidence intervals
HR: hazard ratio
aHR: adjusted hazard ratio
RCTs: randomized controlled trials

## Additional Information and Declarations

### Data Availability

The following information was supplied regarding data availability: The raw measurements are available in the Supplemental Files.
