# Supplementary material for: The association of fracture risk in atrial fibrillation patients and long-term anticoagulant therapy category: a systematic review and meta-analysis
Source: PeerJ. 2021 Jan 25;9:e10683. doi: 10.7717/peerj.10683 (PMC7842143; doi:10.7717/peerj.10683)
Supplement: Supplemental Information 2 [file peerj-09-10683-s002.docx]

***Search strategy：***

**Pubmed:**

**#1:(anticoagulant[MeSH Terms]) OR (direct oral anticoagulant[Title/Abstract]) OR (non-vitamin K antagonist oral anticoagulants[Title/Abstract]) OR ( vitamin K antagonist oral anticoagulants[Title/Abstract])**

**#2:(rivaroxaban[Title/Abstract])OR(apixaban[Title/Abstract])OR(dabigatran[Title/Abstract])OR(edoxaban[Title/Abstract]) OR** **warfarin[Title/Abstract]**

**#3:(fracture[MeSH Terms]) OR(hip fracture) OR (osteoporosis[MeSH Terms]) OR (rarefaction of bone) OR (osteoporotic fracture)**

**#4: #1 OR #2**

**#5: #3 AND #4**

**Embase:**

**#1: 'anticoagulant'/exp OR 'direct oral anticoagulant':ti,ab,kw OR 'non-vitamin k antagonist oral anticoagulants':ti,ab,kw**

**'vitamin K antagonist oral anticoagulants':ti,ab,kw**

**#2: rivaroxaban:ti,ab,kw OR apixaban:ti,ab,kw OR dabigatran:ti,ab,kw OR edoxaban:ti,ab,kw OR warfarin:ti,ab,kw**

**#3: 'fracture'/exp OR 'hip fracture':ti,ab,kw OR osteoporosis:ti,ab,kw OR 'rarefaction of bone':ti,ab,kw OR 'osteoporotic fracture':ti,ab,kw**

**#4: 'atrial fibrillation'/exp**

**#5:#1 OR #2**

**#6: #3 AND #4 AND #5**

**Cochrane Library:**

**#1: MeSH descriptor:[Anticoagulants] explode all trees**

**#2: (direct oral anticoagulant):ti,ab,kw OR (non-vitamin K antagonist oral anticoagulants):ti,ab,kw OR (vitamin K antagonist oral anticoagulants):ti,ab,kw**

**#3:(rivaroxaban):ti,ab,kw OR (apixaban):ti,ab,kw OR (dabigatran):ti,ab,kw OR (edoxaban):ti,ab,kw OR ("Warfarin"):ti,ab,kw**

**#4: (fractures):ti,ab,kw OR ("osteoporosis"):ti,ab,kw OR (rarefaction of bone):ti,ab,kw OR (osteoporotic fractures):ti,ab,kw**

**#5: #1 OR #2 OR #3**

**#6: #4 AND #5**

**Clinical Trials.gov:**

**Condition or disease: atrial fibrillation**

**Other terms: anticoagulant**
