# Supplementary material for: The association of fracture risk in atrial fibrillation patients and long-term anticoagulant therapy category: a systematic review and meta-analysis
Source: PeerJ. 2021 Jan 25;9:e10683. doi: 10.7717/peerj.10683 (PMC7842143; doi:10.7717/peerj.10683)
Supplement: Supplemental Information 3 [file peerj-09-10683-s003.docx]

**The rationale for conducting the systematic review / meta-analysis?**

Meta-analysis refers to use a statistical analysis method to summarizes and analyzes the results of multiple independent studies of the same kind to increase the sample content and improve the test efficiency. In particular, when the results of multiple studies are inconsistent, meta-analysis can be used to get results closer to the real situation.

**The contribution that it makes to knowledge in light of previously published related reports, including other meta-analyses and systematic reviews?**

We were very [appreciate](https://fanyi.so.com/?src=onebox# appreciate) for REZAIEYAZDI^1^ contribution to this field, they [discover](file:///C:/Program%2520Files%2520(x86)/Youdao/Dict/8.9.3.0/resultui/html/index.html#/javascript:;) long-term warfarin therapy was associated with reduced bone density which first revealed the relationship between anticoagulant and bone metabolism that provided [direction](file:///C:/Program%2520Files%2520(x86)/Youdao/Dict/8.9.3.0/resultui/html/index.html#/javascript:;)s for the follow-up related researches. Later, D. Misra et al^2^ made a research warfarin use and risk of osteoporotic fractures based on atrial fibrillation patients, although the results were [negative](file:///C:/Program%2520Files%2520(x86)/Youdao/Dict/8.9.3.0/resultui/html/index.html#/javascript:;), but the academic significance was positive. Veronese et al^3^ made the first research about warfarin use and risk of fractures the first report to demonstrated the relationship between anticoagulant and fractures risk by Meta-Analysis, they argued that warfarin neither increased prospectively-assessed fracture risk compared with healthy controls. However, only two matched studies were pooled for final results, and no analysis across study type or duration of anticoagulation therapy were reported in their study. The results still need to be discussed further. Fiordellisi et al^4^ included 23 articles (22 observational studies and 1 RCT) showed that warfarin use did not increase the odds of fracture, neither for overall fracture nor for specific types of fracture (hip, vertebral, wrist, or rib). In Fiordellisi et al. study, they analysis the subgroup across study types, fracture types, duration of anticoagulation therapy, this provides a novel methodological approach for future research. Recent years, several large studies successively have reported the correlation between anticoagulation and fracture in atrial fibrillation, Lau et al^5^, Binding et al^6^, Lutsey et al^7^, Huang et al^8^ all made the outstanding contributions to this field, those results [reminded](file:///C:/Program%2520Files%2520(x86)/Youdao/Dict/8.9.3.0/resultui/html/index.html#/javascript:;) clinician not just [focus](file:///C:/Program%2520Files%2520(x86)/Youdao/Dict/8.9.3.0/resultui/html/index.html#/javascript:;) on the bleeding adverse events of oral anticoagulants in atrial fibrillation. Also, five early large randomized controlled studies ^9-13^ provided the detailed data for theirs rigorous experimental design and execution. We [appreciate](https://fanyi.so.com/?src=onebox# appreciate) all relevant researchers’ work for this field.

1. Rezaieyazdi Z, Falsoleiman H, Khajehdaluee M, Saghafi M, Mokhtari-Amirmajdi E. Reduced bone density in patients on long-term warfarin. *Int J Rheum Dis*. 2009;12(2):130-135. doi:10.1111/j.1756-185X.2009.01395.x.
2. Misra D, Zhang Y, Peloquin C, Choi HK, Kiel DP, Neogi T. Incident long-term warfarin use and risk of osteoporotic fractures: propensity-score matched cohort of elders with new onset atrial fibrillation. *Osteoporos Int.* 2014;25(6):1677-1684.
3. Veronese N, Bano G, Bertozzo G, Granziera S, Solmi M, Manzato E, et al. 2015. Vitamin K antagonists' use and fracture risk: results from a systematic review and meta-analysis. *J Thromb Haemost* 13:1665-75.
4. Fiordellisi W, White K, and Schweizer M. 2019. A systematic review and meta-analysis of the association between vitamin K antagonist use and fracture. *Journal of general internal medicine* 34:304-311.
5. Lau WC, Chan EW, Cheung CL, et al. Association Between Dabigatran vs Warfarin and Risk of Osteoporotic Fractures Among Patients With Nonvalvular Atrial Fibrillation. *JAMA.* 2017;317(11):1151-1158.
6. Binding C, Bjerring Olesen J, Abrahamsen B, Staerk L, Gislason G, Nissen Bonde A. Osteoporotic Fractures in Patients With Atrial Fibrillation Treated With Conventional Versus Direct Anticoagulants. *Journal of the American College of Cardiology.* 2019;74(17):2150-2158.
7. Lutsey PL, Norby FL, Ensrud KE, et al. Association of Anticoagulant Therapy With Risk of Fracture Among Patients With Atrial Fibrillation. *JAMA Intern Med.* 2019.
8. Huang HK, Liu PP, Hsu JY, et al. Fracture risks among patients with atrial fibrillation receiving different oral anticoagulants: a real-world nationwide cohort study. *Eur Heart J.* 2020.
9. RE-LY study. ClinicalTrials.gov number, NCT00262600.
10. ROCKET AF study. ClinicalTrials.gov number, NCT00403767.
11. ARISTOTLE study. ClinicalTrials.gov number, NCT00412984.
12. J-ROCKET AF study. ClinicalTrials.gov number, NCT00494871.
13. ENGAGE AF-TIMI 48 study. ClinicalTrials.gov number, NCT00781391.
