# Supplementary material for: The association of fracture risk in atrial fibrillation patients and long-term anticoagulant therapy category: a systematic review and meta-analysis
Source: PeerJ. 2021 Jan 25;9:e10683. doi: 10.7717/peerj.10683 (PMC7842143; doi:10.7717/peerj.10683)
Supplement: Supplemental Information 5 [file peerj-09-10683-s005.docx]

***Supplement table1: The fractures numbers of each RCTs.***

|  | **Patel/2011** | | **Connolly/2009** | | | **Granger/2011** | | **Giugliano/2013** | | | **Hori/2012** | |
| --- | --- | --- | --- | --- | --- | --- | --- | --- | --- | --- | --- | --- |
| **Characteristic** | **Rivaroxaban** | **Warfarin** | **Dabigatran**  **110mg** | **Dabigatran**  **150mg** | **Warfarin** | **Apixaban** | **Warfarin** | **Edoxaban**  **60mg** | **Edoxaban**  **30mg** | **Warfarin** | **Rivaroxaban** | **Warfarin** |
| **Size** | **7111** | **7125** | **5983** | **6059** | **5998** | **9052** | **9088** | **7012** | **7002** | **7012** | **639** | **639** |
| **all-fractures** | **82** | **115** | **44** | **43** | **34** | **147** | **119** | **199** | **212** | **227** | **9** | **11** |
| **hip-fractures** | **15** | **16** | **8** | **8** | **3** | **28** | **19** | **19** | **29** | **28** | **NA** | **NA** |

***Supplement table2: The fractures risk of each RCTs (fracture risk ratio of each NOACs vs warfarin)***

|  | **Patel/2011** | **Connolly/2009** | | **Granger/2011** | **Giugliano/2013** | | **Hori/2012** |
| --- | --- | --- | --- | --- | --- | --- | --- |
| **Characteristic** | **Rivaroxaban**  **vs warfarin** | **Dabigatran**  **110mg**  **vs warfarin** | **Dabigatran**  **150mg**  **vs warfarin** | **Apixaban**  **vs warfarin** | **Edoxaban**  **60mg**  **vs warfarin** | **Edoxaban**  **30mg**  **vs warfarin** | **Rivaroxaban**  **vs warfarin** |
| **all-****fractures** | **0.71**  **[0.54-0.95]** | **1.30**  **[0.83-2.03]** | **1.25**  **[0.80-1.96]** | **1.24**  **[0.98-1.58]** | **0.88**  **[****0.73-1.06]** | **0.94**  **[0.78-1.12]** | **0.82**  **[0.34-1.98]** |
| **hip-****fractures** | **0.94**  **[0.46-1.90]** | **2.67 [0.71-10.06]** | **2.63**  **[0.70-9.94]** | **1.41**  **[0.80-2.50]** | **0.68**  **[0.38-1.21]** | **1.03**  **[0.62-1.74]** | **NA** |

***Supplemental table3: The risk bias of each RCTs：***

|  | **Sequence generation** | **Blinding of**  **participants and personnel** | **Allocation sequence concealment** | **Blinding of**  **outcome assessment** | **Selective outcome reporting** | **Incomplete of outcome data** | **other threats to validity** |
| --- | --- | --- | --- | --- | --- | --- | --- |
| **Connolly/2009** | **Low risk** | **Low risk** | **Low risk** | **Low risk** | **Low risk** | **Low risk** | **Low risk** |
| **Patel/2011** | **Low risk** | **Low risk** | **Low risk** | **Low risk** | **Low risk** | **Low risk** | **Low risk** |
| **Granger/2013** | **Low risk** | **Low risk** | **Low risk** | **Low risk** | **Low risk** | **Low risk** | **Low risk** |
| **Hori/2012** | **Low risk** | **Low risk** | **Low risk** | **Low risk** | **Low risk** | **Low risk** | **Low risk** |
| **Giugliano/2013** | **Low risk** | **Low risk** | **Low risk** | **Low risk** | **Low risk** | **Low risk** | **Low risk** |

***Supplemental table4: The risk bias of each observational studies：***

|  | **Representativeness of the exposed cohort** | **Selection of the non exposed cohort** | **Ascertainment of exposure** | **Outcome not present at start of study** | **Basis between comparisons** | **Assessment of outcome** | **follow-up enough for outcomes to occur** | **Adequacy of follow up of cohorts** | **Total points** |
| --- | --- | --- | --- | --- | --- | --- | --- | --- | --- |
| **Binding/2019** | **Some representative** | **same community** | **secure record** | **yes** | **control** | **record linkage** | **enough** | **yes** | **8** |
| **Patel/2011** | **Some representative** | **same community** | **secure record** | **yes** | **control** | **record linkage** | **enough** | **yes** | **8** |
| **Granger/2013** | **Some representative** | **same community** | **secure record** | **yes** | **control** | **record linkage** | **enough** | **yes** | **8** |
| **Hori/2012** | **Some representative** | **same community** | **secure record** | **yes** | **control** | **record linkage** | **enough** | **yes** | **8** |
| **Lucenteforte/2017** | **Larger bias** | **same community** | **secure record** | **yes** | **Not control** | **record linkage** | **enough** | **no statement** | **5** |
