# Supplementary figures and images for: The association of fracture risk in atrial fibrillation patients and long-term anticoagulant therapy category: a systematic review and meta-analysis
Source: PeerJ. 2021 Jan 25;9:e10683. doi: 10.7717/peerj.10683 (PMC7842143; doi:10.7717/peerj.10683)

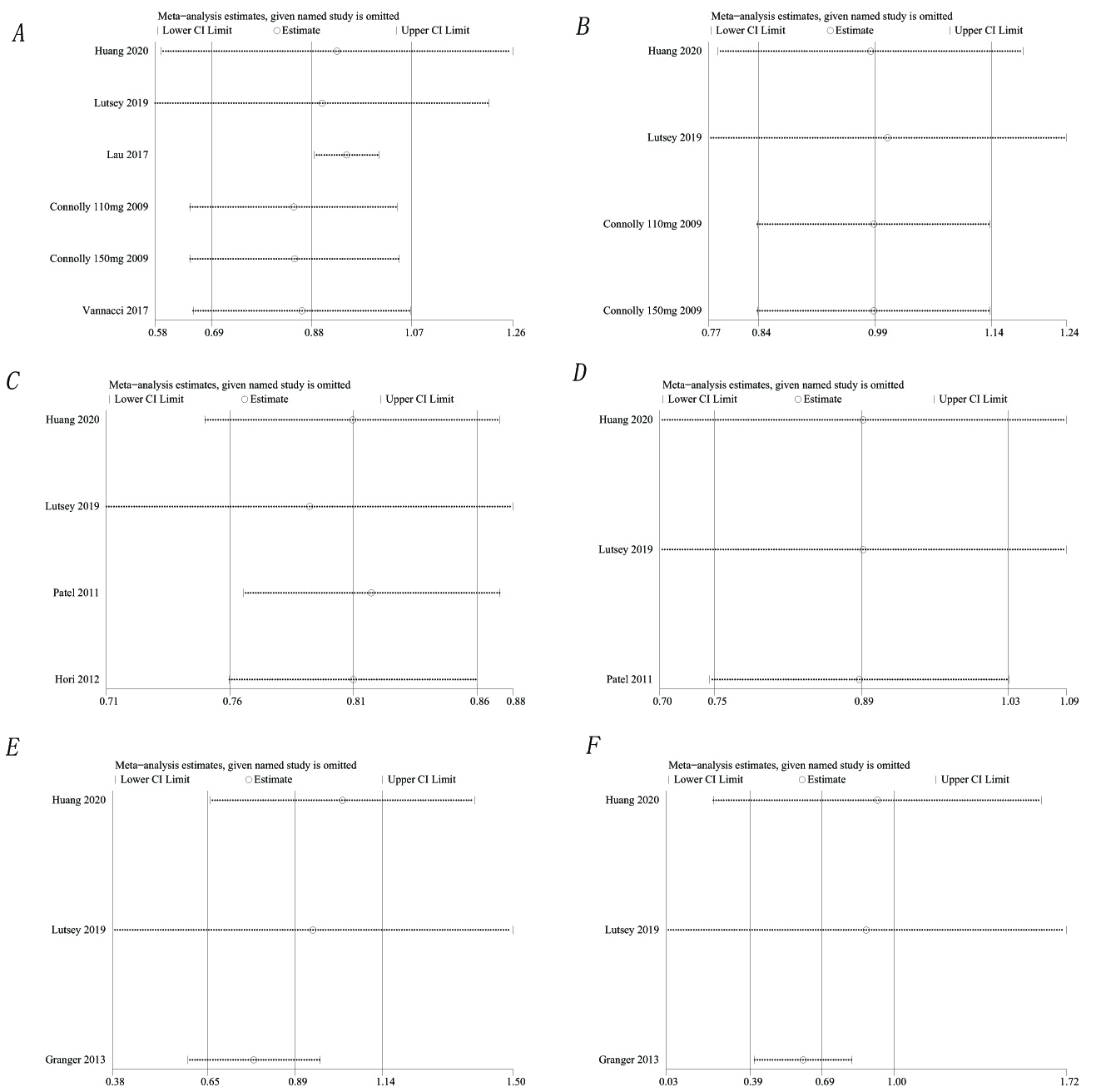

Supplement: Supplemental Information 6 — (A) The sensitivity analysis for all fracture risk of dabigatran versus warfarin; (B) The sensitivity analysis for hip fracture risk of dabigatran versus warfarin; (C) The sensitivity analysis for all fracture risk of rivaroxaban versus warfarin; (D) The sensitivity analysis for hip fracture risk of rivaroxaban versus warfarin; (E) The sensitivity analysis for all fracture risk of apixaban versus warfarin; (F) The sensitivity analysis for hip fracture risk of apixaban versus warfarin. [file peerj-09-10683-s006.jpg]
